# Gut microbiota composition alterations are associated with the onset of diabetes in kidney transplant recipients

**DOI:** 10.1371/journal.pone.0227373

**Published:** 2020-01-07

**Authors:** Marie Lecronier, Parvine Tashk, Yanis Tamzali, Olivier Tenaillon, Erick Denamur, Benoit Barrou, Judith Aron-Wisnewsky, Jérôme Tourret

**Affiliations:** 1 INSERM, IAME, UMR 1137, Université Paris Diderot, Sorbonne Paris Cité, Paris, France; 2 AP-HP, Département d’Urologie, Néphrologie et Transplantation, GH Pitié-Salpêtrière Charles Foix, Paris, France; 3 AP-HP, Laboratoire de Génétique Moléculaire, Hôpital Bichat, Paris, France; 4 Sorbonne Université, Paris, France; 5 AP-HP, Institute of Cardiometabolism and Nutrition, ICAN, Service de nutrition, GH Pitié-Salpêtrière Charles Foix, Paris, France; 6 INSERM, UMR_S U1166, équipe NutriOmics, Paris, France; Western University of Health Sciences, UNITED STATES

## Abstract

Background

The gut dysbiosis associated with diabetes acquired before or after kidney transplantation (KT) has not been explored.

**Methods:**

Patients transplanted at our institution provided fecal samples before, and 3–9 months after KT. Fecal bacterial DNA was extracted and 9 bacteria or bacterial groups were quantified by qPCR.

**Results:**

50 patients (19 controls without diabetes, 15 who developed New Onset Diabetes After Transplantation, NODAT, and 16 with type 2 diabetes before KT) were included. Before KT, *Lactobacillus sp*. tended to be less frequently detected in controls than in those who would become diabetic following KT (NODAT) and in initially diabetic patients (60%, 87.5%, and 100%, respectively, p = 0.08). The relative abundance of *Faecalibacterium prausnitzii* was 30 times lower in initially diabetic patients than in controls (p = 0.002). The relative abundance of *F*. *prausnitzii* of NODAT patients was statistically indistinguishable from controls and from diabetic patients. The relative abundance of *Lactobacillus sp*. increased following KT in NODAT and in initially diabetic patients (20-fold, p = 0.06, and 25-fold, p = 0.02, respectively). In contrast, the proportion of *Akkermansia muciniphila* decreased following KT in NODAT and in initially diabetic patients (2,500-fold, p = 0.04, and 50,000-fold, p<0.0001, respectively). The proportion of *Lactobacillus* and *A*. *muciniphila* did not change in controls between before and after the transplantation. Consequently, after KT the relative abundance of *Lactobacillus sp*. was 25 times higher (p = 0.07) and the relative abundance of *A*. *muciniphila* was 2,000 times lower (p = 0.002) in diabetics than in controls.

**Conclusion:**

An alteration of the gut microbiota composition involving *Lactobacillus sp*., *A*. *muciniphila* and *F*. *prausnitzii* is associated with the glycemic status in KT recipients, raising the question of their role in the genesis of NODAT.

## Introduction

Many diseases and disorders, including obesity and diabetes, have been linked to a change in the composition of the gut microbiota called dysbiosis [1], both in mouse models and in humans. Microbial diversity is dramatically decreased in obese patients with metabolic disorders compared to obese patients without [2–6]. The Firmicutes to Bacteroidetes phyla ratio is increased in obese mice and patients [3, 7]. In addition, in obese or diabetic patients, *Bifidobacterium* and *Faecalibacterium prausnitzii* [8] are decreased and *Bacteroides* and *Lactobacilli* [9] are increased [10, 11]. Finally, the lower proportion of *F*. *prausnitzii* in diabetic patients is restored after weight loss and metabolic improvement either with diet intervention [12] or after bariatric surgery [13]. *Akkermansia muciniphila* has been associated with insulin sensitivity. Indeed, obese mice fed with prebiotic carbohydrates show clinical benefit including weight loss and improved insulin sensitivity [14]. Furthermore, gavage of obese mice with live or pasteurized *Akkermansia muciniphila* recapitulated these beneficial effects [15–17]. Finally, we also confirmed that among overweight or obese individuals the proportion of *Akkermansia muciniphila* was higher in insulin-sensitive patients [18].

Microbiota transfer experiments in germ-free mice suggest that dysbiosis is not only associated with, but also responsible for these metabolic disorders [19–22]. Importantly, fecal transplantation from lean humans to metabolically affected obese individuals induced a significant improvement in insulin sensitivity, associated with a modification in gut microbiota composition with an increase in *Akkermansia muciniphila* [23, 24].

Metabolic disorders are very common in kidney transplant (KT) recipients (KTRs), both before and after the transplantation. Diabetes is the first cause for end-stage renal disease and the requirement for KTR worldwide, with approximately 40% of diabetics on the waiting list [25]. In addition, normoglycemic patients before KT are at increased risk of new onset diabetes after transplantation (NODAT; [26]) which develops in approximately 20% of KTRs in the first year after transplantation [27, 28]. This is mainly due to the immunosuppressive (IS) treatment, which may include corticosteroids [29], cyclosporin, tacrolimus [30] and sirolimus [31] which have been shown to induce either insulin resistance or alteration in insulin secretion. In turn, this worsening metabolic syndrome negatively impacts the outcome of KTRs in terms of cardiovascular and renal events [26].

The possible role of the gut microbiota in the genesis of diabetes before or after KT remains to be explored. However, the interactions between metabolic disorders, the microbiota, and IS drugs are highly complex in the context of KT. Indeed, a dysbiosis and a “leaky gut” have been described in chronic kidney disease patients [32, 33], and IS drugs significantly alter microbiota composition [34].

We decided to investigate whether alterations in the gut microbiota composition observed in diabetic patients in the general population were also associated with diabetes before and/or after KT. For this purpose, we measured the relative abundance of nine bacteria or bacterial groups that have been shown to be associated to metabolic disorders in the feces collected before and after KT in initially diabetic, NODAT and control KTRs.

## Material and methods

### Study population and definitions of diabetes and NODAT

Between September 2013 and December 2014, we prospectively collected feces from all patients undergoing transplantation at our institution. All patients admitted for a kidney transplantation were required to provide a fecal sample upon arrival in the department, before administration of any immunosuppressive drug (“D0 sample”), and 3 months after the KT. Due to organizational reasons (both from the patients’ and the staff’s sides) the second samples were in reality collected 3 to 9 months after KT and the samples are designated as “M3-9 samples” in the manuscript.

Patients receiving a combined KT (kidney and liver, or kidney and heart transplantations) were excluded because they were treated and followed by different medical teams.

The only inclusion criterion for this study was that the D0 sample and/or the M3-9 sample needed to contain enough material to allow a DNA extraction of at least 500 ng.

Type 2 diabetes and New Onset Diabetes After Transplantation (NODAT) were defined according to the diagnostic criteria of the American Diabetes Association [35], except that no oral glucose tolerance test was performed.

### Ethical considerations

This study was approved by the local ethic committee (“CPP Ile de France VI”) on 28 March 2013. All patients were orally informed of the research upon arrival in our institution to receive a kidney transplant. They were free to refuse to provide stool samples if they did not want to participate. No written consent was required by local authorities, as the present research did not modify the usual follow-up of transplanted patients in our institution.

### Therapeutic protocol for transplanted patients

The proportion of patients who received basiliximab or anti-lymphocyte polyclonal antibody induction was not different in controls, NODAT and diabetic patients (Table 1).

**Table 1:** Induction therapy, valganciclovir and antibiotic use after kidney transplantation.

	Controls (N = 19), %	NODAT (N = 15), %	Diabetics (N = 16), %	Total (N = 50), %	P^a^
Induction therapy					
Polyclonal Anti-Lymphocyte Ig	77	93	80	83	0.5
Basiliximab	24	7.1	20	11	
Valganciclovir for the treatment of CMV infection before M3-9 sample	58	87	73	71	0.2
Antibiotic treatment before M3-9 sample (other than antibioprophylaxis)	21	36	40	31	0.5

Ig: immunoglobulin

^a^The P value shown in the table is the result of X^2^ tests. CMV: cytomegalovirus.

The initial maintenance immunosuppressive regimen in our institution always consists of a combination of prednisone (20 mg QD progressively tapered to 5 mg QD between the 4^rd^ and the 9^th^ month), tacrolimus (target trough level 8±2 ng/ml) and mycophenolate mofetil (dose adapted in order to obtain an estimated area under the curve around 30 h x mg/l). We had no clinical indication to change this initial regimen in any of the included patients for the total duration of this study. All patients received cotrimoxazole for pneumocystis prophylaxis during the total duration of this study. Valgancyclovir treatment was prescribed only to patients who presented a CMV infection (systematic CMV qPCR during follow-up) or disease. The proportion of valgancyclovir and antibiotic treatments initiated between D0 and the time of collection of the M3-9 sample was not different between the three groups (Table 1).

### Fecal swabs

In all cases, we used swabs (modified Cary-Blair medium FecalSwab^®^, Copan^®^, Milan, Italy) either to harvest a sample from spontaneously emitted feces or to perform a rectal swabbing (when patients could not provide feces before surgery at D0). Samples were stored at 4°C for a maximum of 2 days before they were frozen at -30°C [36].

### DNA extraction

Fecal samples were thawed, and 400 μl of the feces and storage medium mix were centrifuged (8,000 rpm, 8 min). The fecal DNA was extracted from the pellet with the QIAamp DNA Stool Mini Kit^®^ (Qiagen^®^) following the manufacturer’s instructions, with the addition of an initial 1-min bead-beating step on a FastPrep-24 (MP Biomedicals, Solon, OH) on level 5. The quality and quantity of the DNA collected were assessed on a Nanodrop^®^ analyzer. All DNA samples were kept at -80°C until use.

### qPCR quantification of fecal bacterial groups

The fecal microbiota from KTRs was explored at various taxonomic levels through the quantification of nine bacterial groups or species by qPCR: The Firmicutes/Bacteroidetes ratio, *Bacteroides-Prevotella* group, *Lactobacilli*, *Bifidobacteria*, *Akkermansia muciniphila*, *Faecalibacterium prausnitzii*, *Escherichia coli*, *Clostridium coccoides*, and *Clostridium leptum*.

All these bacteria or bacterial groups were specifically chosen as they have been shown to be associated with metabolic disorders in mice and/or the population of non-transplanted patients.

After extraction, fecal bacterial DNA was quantified using a Nanodrop^®^ analyzer and diluted in order to obtain a concentration of 10 ng/μl. qPCRs included the template (50 ng of DNA per reaction), 5 μL of 2X SYBR Green mix (Absolute blue^®^ qPCR SYBR Green, Thermo scientific^®^, including Taq hot start enzyme) and each primer to a final concentration in the mix of 300 nM. Water was added to obtain a final reaction volume of 10 μL. The sequence, and annealing temperature of primer pairs used to quantify each bacterium or bacterial group is shown in S1 Table. qPCRs were carried out in a LightCycler480 (Roche^®^) as follows: one initial activation step of 15 min at 95°C, 40 cycles of 2-step amplifications (95°C for 15” for denaturation, 57–63°C for 1 min for annealing). A bacterium or a bacterial group was considered undetectable in a sample if its quantification cycle (Cq) was ≥ 35.

All qPCRs were followed by a dissociation curve to check for the amplification of a unique DNA fragment.

All bacterial quantifications were performed on two independent qPCRs, each containing a duplicate of each sample in the same 96 well plate, with the result calculated as the mean of the 4 measures. Template DNA was thawed only twice, once for each repeat of the qPCR.

The relative amount of a bacterium or a bacterial group in a given sample was inferred with the following formula:

$$Q_{Bac}=Log_{10}(2^{CqEub‐CqBac})$$

where Q_Bac_ is the (log-) relative abundance of the studied bacterium or bacterial group in the sample. The formula is based on the qPCR cycle number (Cq) where the SYBR Green signals exceed the detection threshold: CqEub is the mean Cq obtained with the “Eubacteria” pair of primers (see S1 Table) which quantifies all bacteria in the sample, and CqBac is the mean Cq obtained with the pair of primers specific of the studied bacterium or bacterial group.

### Statistical analysis

Quantitative data are presented as mean ± standard deviation (normally distributed data) or as median [interquartile range] (variables without a normal distribution). The difference between the means of more than two groups was tested by one-way ANOVA (normally distributed data) or Kruskal-Wallis ANOVA (variables without a normal distribution). When these ANOVA estimates produced a significant difference (p<0.05), Turkey’s test with correction for multiple comparisons was used to identify which means in the series were different from one another. If the groups which were compared contained only paired data (measures in the same patients at two time points), repeated measures ANOVA was used.

The difference between the means of several groups at two time points (D0 and M3-9) was estimated by two-way ANOVA. When two-way ANOVA showed a significant difference (p<0.05), Turkey’s test with correction for multiple comparisons was used to identify which means in the series were different from one another.

The difference between qualitative data was tested by X^2^ tests.

All statistical tests were performed by GraphPad Prism^®^ v6.05, San Diego, CA, USA.

## Results

### Patients’ characteristics

**Fig 1:**
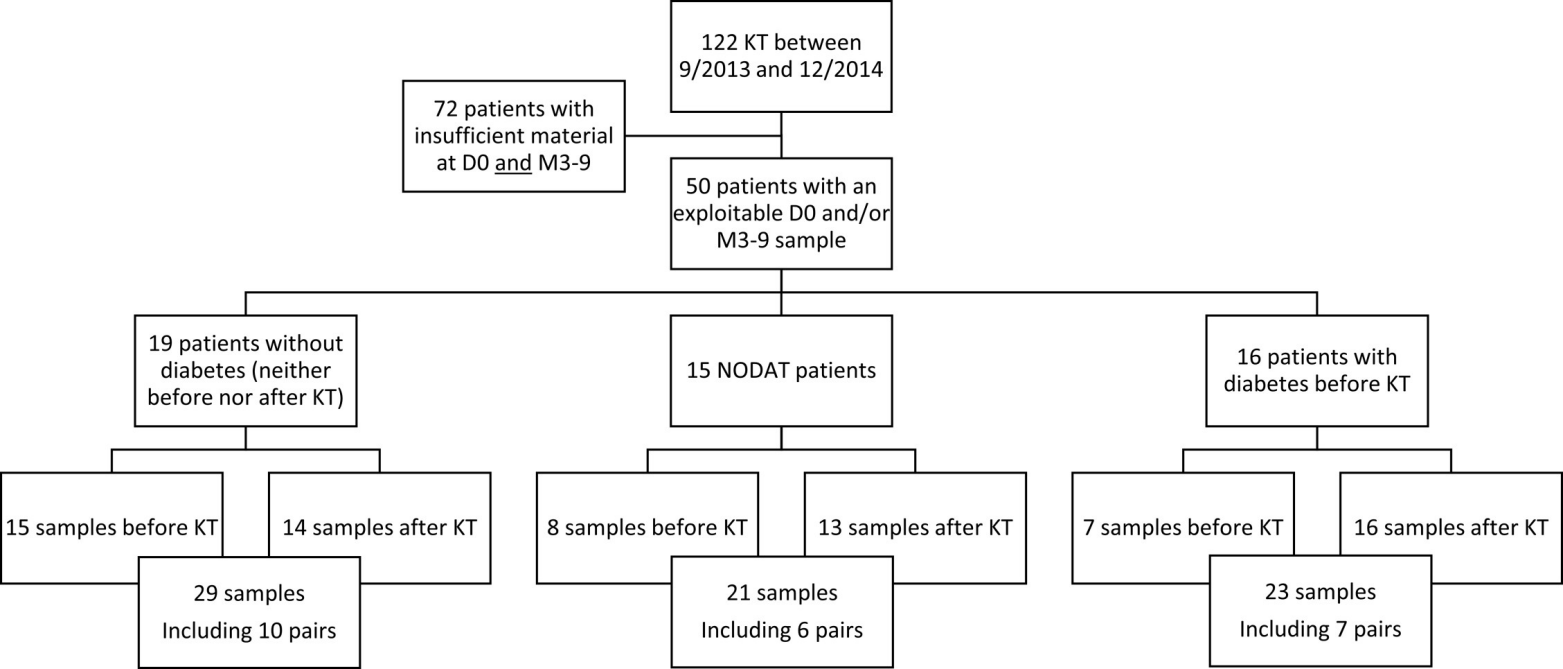
Flowchart indicating the patients who were included and the samples that they provided. Out of 122 KTRs, 50 patients (19 non-diabetic controls, 15 NODAT patients and 16 initially diabetic patients) provided 73 samples. NODAT: New Onset Diabetes After Transplantation.

Fifty patients met the inclusion criterion: 19 were non-diabetic and non-obese both before and after KT (controls), 15 developed New Onset Diabetes After Transplantation (NODAT) and 16 were initially diabetic patients (Fig 1).

All included patients received a kidney transplant (KT) between September 2013 and December 2014. Over the same period, we performed 122 KT at our institution. Unfortunately, the amount of feces recovered after rectal swabbing was often insufficient and did not allow a satisfactory extraction of bacterial DNA. For this reason, only 50 patients out of the 122 patients were enrolled.

In the overall cohort, mean age was 54.9±12 years, 66% were males and 28% originated from Sub Saharan Africa (Table 2).

**Table 2:** Patient characteristics.

	Controls (N = 19) Mean ± SD or N (%)	NODAT (N = 15) Mean ± SD, or N (%)	Diabetics (N = 16) Mean ± SD, or N (%)	Total (N = 50) Mean ± SD, or N (%)	P^b^
Age, years	51.2±13.5	56.3±11.4	58.1±9.4	54.9±11.8	0.20
Sex, males	11 (58)	9 (60)	13 (81.3)	33 (66)	0.29
Ethnicity					0.55
Sub-Saharan Africa	4 (21.0)	4 (26.7)	6 (37.5)	14 (28)	
Other	15 (79)	11 (67.3)	10 (62.5)	36 (72)	
Initial nephropathy					**<0.0001**
Diabetic nephropathy	0 (0)	0 (0)	14 (87.5)	14 (28)	
Hypertensive kidney disease	2 (10.5)	3 (20)	1 (6.3)	6 (12)	
Polycystic kidney disease	3 (15.7)	7 (46.7)	0 (0)	10 (20)	
Undetermined	4 (21.1)	1 (6.7)	0 (0)	5 (10)	
Other	10 (52.6)	4 (26.7)	1 (6.3)	15 (30)	
History of diabetes mellitus	0 (0)	0 (0)	16 (100)	16 (32)	
Dialysis duration, years	2.2±1.9	3.4±2.6	3.3±2.0	3.0±2.2	0.08
HbA1c before KT, %	5.5±0.5	6.0±0.4	7.0±2.2	6.2±1.5	**<0.0001**
Obesity before KT^a^	0 (0)	3 (20)	4 (25)	7 (14)	0.08
BMI at KT, kg/m^2^	23.6±3.2	26.6±4.7	26.6±3.4	25.2±3.9	**0.0004**
HbA1c after KT, %	5.5±0.6	6.8±0.9	7.3±1.1	6.5±1.2	**<0.0001**
BMI max after KT, kg/m^2^	24.2±2.9	27.0±4.9	28.0±4.3	26.2±4.3	**<0.05**
Nadir of creatinine, μmol/l	112.0±38	106±67	107±25	108±48	0.82

^a^Obesity is defined as a body mass index ≥30kg/m^2^

BMI: Body mass index, KT: kidney transplantation

^b^The P value shown in the table is the result of two-way ANOVA, or X^2^ test (qualitative data).

**Fig 2:**
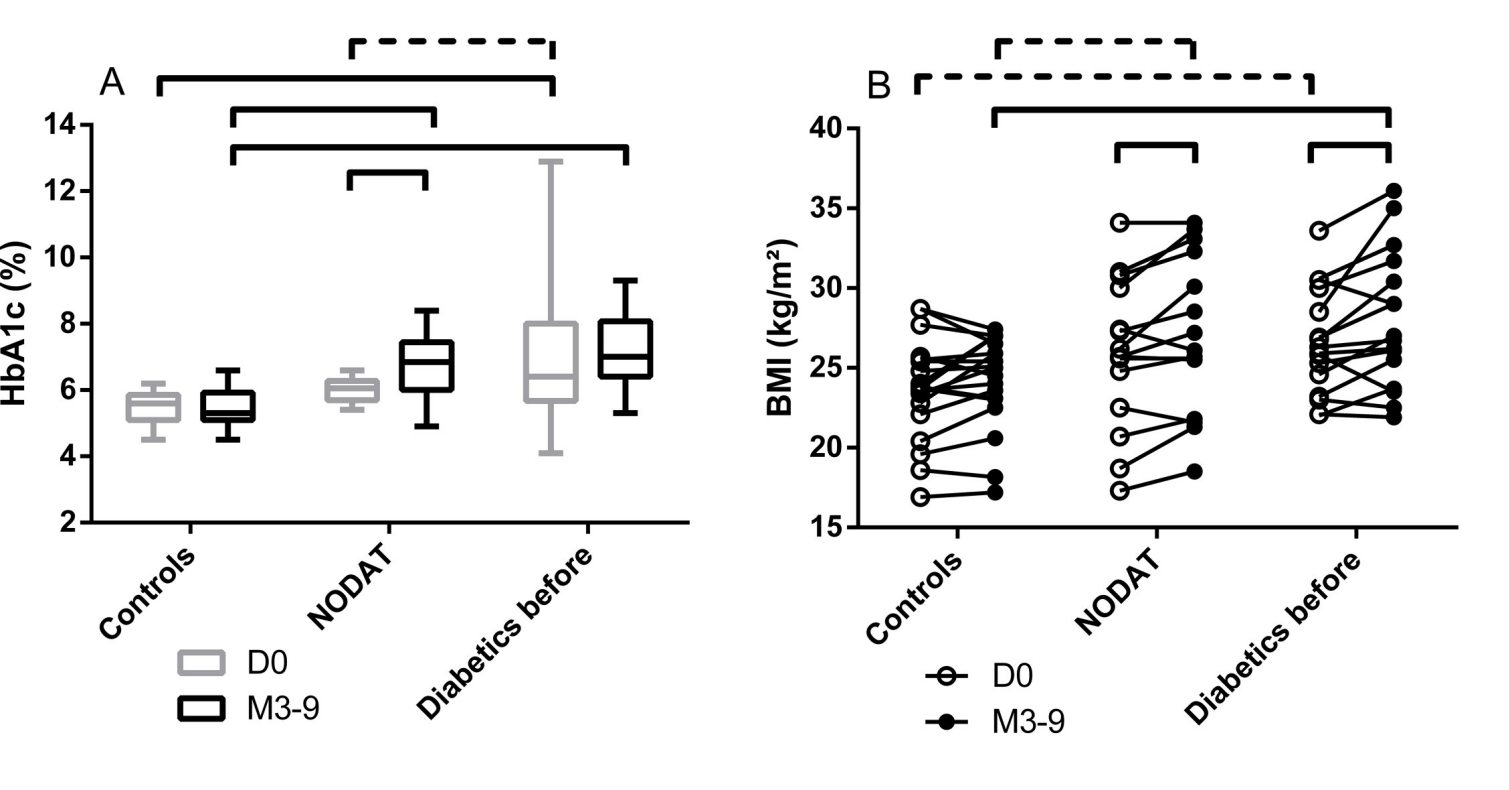
Glycated hemoglobin (A) and Body Mass Index (B) of included patients, before and after transplantation according to their diabetic status. Bars indicate significant differences and dotted bars indicate trends (p<0.1) between groups (standard one-way ANOVA). Number of patients: (A) D0: Controls, n = 17; NODAT, n = 12; diabetics before transplantation, n = 16. M3-9: Controls, n = 18; NODAT, n = 14; diabetics before transplantation, n = 17. (B): no missing data both at D0 and M3-9: Controls, n = 19; NODAT, n = 15; diabetics before transplantation, n = 16. NODAT: New Onset Diabetes After Transplantation.

As expected, HbA1c levels at inclusion were different in the three groups (p<0.0001 for the metabolic group by two-way ANOVA, Fig 2). At D0, HbA1c was higher in initially diabetic patients than in controls (7.0±2.1% vs. 5.5±0.5%, p = 0.0005 by two-way ANOVA with Turkey’s correction for multiple comparisons) and tended to be higher in diabetics than in NODAT patients (7.0±2.1% vs. 6.0±0.3%, p = 0.06, same method).

The Body mass index (BMI) was also different in the three groups before KT (p = 0.03 for the metabolic group by two-way ANOVA). The BMI tended to be higher in diabetic patients than in controls at inclusion (26.6±3.4 kg/m^2^ vs. 23.6±3.2 kg/m^2^, p = 0.06 by two-way ANOVA with Turkey’s correction).

The sex ratio (66% vs. 87.8%, p = 0.5), and the mean age (60.3±11.4 years vs. 56.4±15.0 years, p = 0.12), BMI (25.3±3.8 kg/m^2^ vs. 25.1±35 kg/m^2^, p = 0.8), HbA1c (8.0±11.3% vs. 8.0±10.4%, p = 1), and dialysis duration (35.8±26.3 months vs. 47.7±55.4 months, p = 0.2) were not different between the 50 patients included in this study and the 72 other patients transplanted over the same period in our institution who did not provide enough feces to be included.

Three to nine months after transplantation, HbA1c levels were lower in controls (5.5±0.6%), than in NODAT patients (6.8±1%, p<0.004 by two-way ANOVA with Turkey’s correction), and in initially diabetic patients (7.3±1.0% p<0.0001; Table 2 and Fig 2). Consistent with the patients’ selection, HbA1c significantly increased in NODAT patients between before and after KT (6.0±0.3% *vs*. 6.8±0.9%, p = 0.01 by t-test with Bonferroni’s correction for multiple comparisons) and reached the level of Hba1c of initially diabetic patients (p = 0.45).

Controls had a lower BMI than diabetic patients (24.2±2.9 kg/m^2^ vs. 28.0±4.3 kg/m^2^, p = 0.01 by repeated measures two-way ANOVA with Turkey’s correction) and tended to have a lower BMI than NODAT patients (24.2±2.9 kg/m^2^ *vs*. 27.0±4.9 kg/m^2^, p = 0.09, respectively, same method).

### Collected fecal samples

A total of 73 fecal samples were collected from 50 patients, as we were not able to collect a sample both before and after KT from all patients (Fig 1). The samples collected before KT were all obtained 24 hours before KT, before any immunosuppressive or antibiotic treatment was started. Therefore, these samples are referred to as “D0 samples”. Samples collected after transplantation were collected after a median delay of 3.3 [3.1–7.8] months for controls, 3.1 [2.8–7.7] months for NODAT patients, and 3.3 [3.0–5.5] months for initially diabetic patients (p = 0.65 by Kruskal-Wallis ANOVA). Because of this delay of approximately 3 to 9 months after KT, post-KT samples are referred to as “M3-9 samples”.

### Fecal bacteria species associated with NODAT and diabetes prior to KT

**Fig 3:**
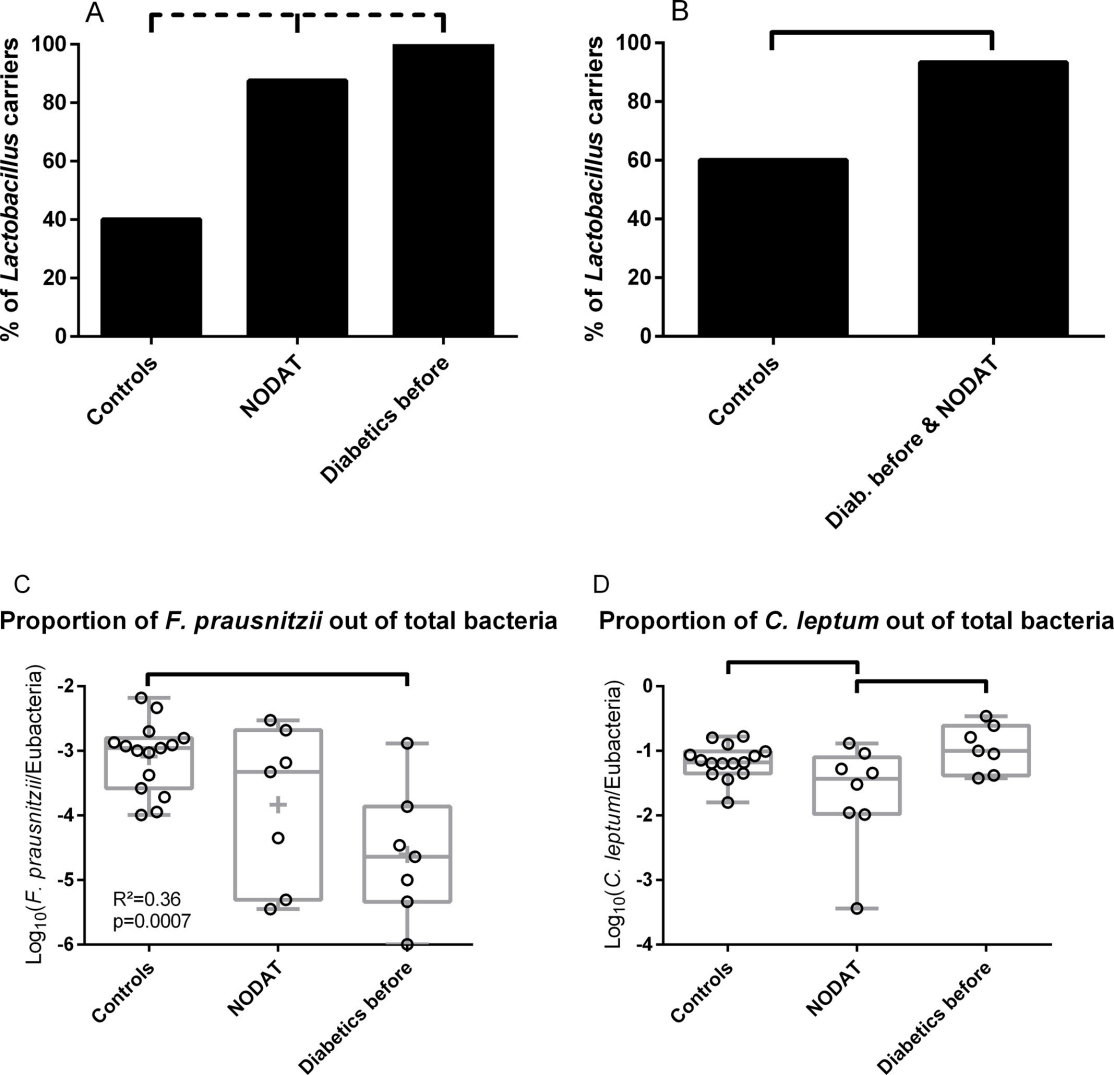
Quantification of fecal bacteria species before kidney transplantation. A, B: Quantification of *Lactobacilli* in fecal samples collected before kidney transplantation. The proportion of patients harboring *Lactobacilli* in their feces before KT tended to be higher in NODAT patients and in initially diabetic patients than in controls (A; p = 0.08 by X^2^ test). When initially diabetic and NODAT patients were grouped together, they more frequently harbored *Lactobacilli* in their feces than controls (B; p = 0.03 by X^2^ test). C: Relative abundance of *Faecalibacterium prausnitzii* out of the total bacteria in the feces of transplanted patients collected before KT. Initially diabetic patients had a lower proportion of *F*. *prausnitzii* than controls. NODAT harbored an intermediate relative abundance of *F*. *prausnitzii* in their feces. D: Relative abundance of *Clostridium leptum* out of the total bacteria in the feces of transplanted patients collected before KT. Bars at the top of graphs indicate significant differences; dotted bars at the top of the graph indicate trends (p≤0.08); A, B: X^2^ test. C, D: One-way ANOVA. NODAT: New Onset Diabetes After Transplantation.

Before KT, *Lactobacillus sp*. was detected in 60% of the feces from controls, while it was always detected in the feces of initially diabetic patients. At D0, patients who would develop diabetes after KT (NODAT patients) tended to have an intermediate rate of carriage of *Lactobacillus sp*. (87.5%, X^2^ test for the comparison of the three groups: p = 0.08; Fig 3A). When initially diabetic patients were grouped with the patients who would become diabetic after KT (NODAT patients), the proportion of patients in whom *Lactobacillus* was detected was significantly higher than in controls (93.3% *vs*. 60%, p = 0.03; Fig 3B).

At the species level, the relative abundance of *Faecalibacterium prausnitzii* was significantly different in the feces of the three groups of patients at D0 (p = 0.002 by one-way ANOVA, Fig 3C). The relative abundance of *F*. *prausnitzii* was significantly lower in the feces of patients who were diabetic before KT compared to controls (difference between the means of Log_10_(*F*. *prausnitzii*/Eubacteria) = -1.5, *i*.*e*. the relative abundance of *F*. *prausnitzii* was 30 times lower in the diabetics than in controls, p = 0.002 by one-way ANOVA with Turkey’s correction for multiple comparisons).

*Clostridium leptum* was also unequally distributed in the three groups of patients (p = 0.02 by one-way ANOVA, Fig 3D). The relative abundance of *C*. *leptum* in NODAT patients was one third the proportion in controls (p = 0.05 by one-way ANOVA with Turkey’s correction), and one fifth the proportion in initially diabetic patients (p = 0.02).

In contrast, we did not observe any significant differences in the Firmicutes/Bacteroidetes ratio, the presence and the relative abundance of *Bifidobacterium sp*., *Bacteroides-Prevotella* group, *Akkermansia muciniphila*, *Escherichia coli*, and *Clostridium coccoides* between the three groups of patients at D0.

To summarize, a higher probability of detectable *Lactobacillus sp*. in the feces and a lower relative abundance of *F*. *prausnitzii* characterized the gut microbiota alteration associated with the initial diabetes and (ulterior) NODAT at D0, as compared to the controls.

### Fecal bacteria species associated with NODAT and diabetes after KT

**Fig 4:**
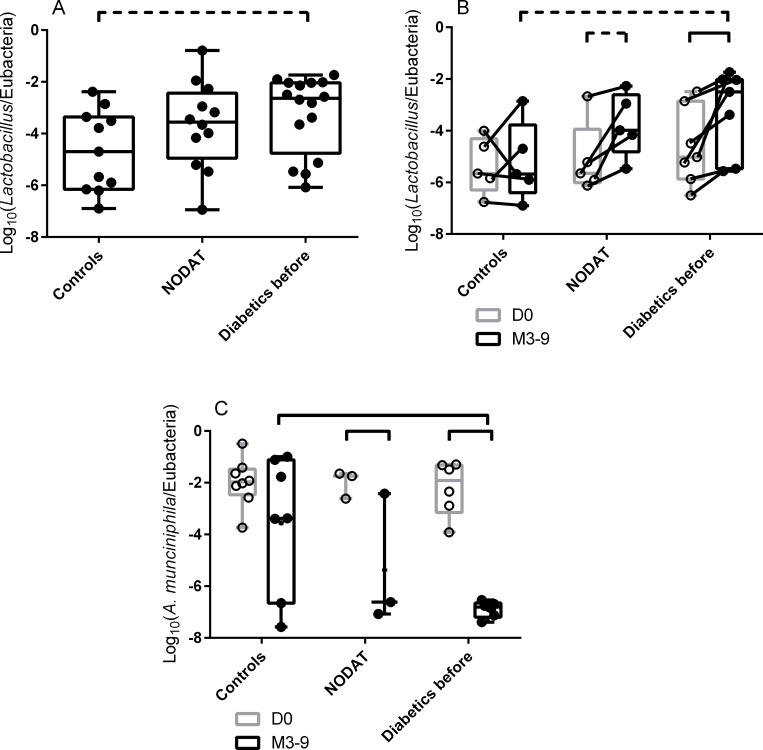
Quantification of specific fecal bacteria species 3 to 9 months after kidney transplantation. A: Quantification of *Lactobacilli* in fecal samples collected 3–9 months after kidney transplantation. The relative abundance of *Lactobacilli* out of the total bacteria was higher in diabetic patients than in controls. NODAT patients had an intermediate proportion of *Lactobacilli*. B: Relative abundance of *Lactobacilli* in fecal samples collected at D0 and 3–9 months after kidney transplantation, restricted to carriers for whom a sample at D0 *and* 3–9 months after kidney transplantation was available (paired samples from the same patients before and after KT). The relative abundance of *Lactobacilli* tended to increase between D0 and M3-9 in NODAT patients and increased in patients who were diabetics before KT. Finally, the relative abundance of *Lactobacilli* tended to be higher in diabetic patients than in controls at M3-9. C: Relative abundance of *Akkermansia muciniphila* in fecal samples collected at D0 and 3–9 months after kidney transplantation. The relative abundance of *A*. *muciniphila* out of the total bacteria decreased between D0 and M3-9 in NODAT and diabetic patients. In addition, it was lower in diabetic patients than in controls at M3-9. Finally, NODAT patients had an intermediate relative abundance of *A*. *muciniphila*. Box (25th to 75th percentiles) and whiskers (min to max) with the median (line in the middle) and all individual values (rounds) are plotted. Bars at the top of graphs indicate significant differences. Dotted bars indicate trends (p≤0.08).

Three to nine months after KT, at the genus level, *Lactobacillus sp*. was detected in almost all diabetics (*i*.*e*. initially diabetic patients and NODAT patients, 96.6%), while it was detected in fewer controls (78.6%, p = 0.05) in M3-9. Furthermore, the relative abundance of *Lactobacillus sp*. in the feces of patients from the three groups tended to be different in M3-9 samples (p = 0.07 by one-way ANOVA, Fig 4A). This relative abundance tended to be higher in initially diabetic patients compared to controls (25-fold, p = 0.06 by one-way ANOVA with Turkey’s correction). NODAT patients had an intermediate proportion of *Lactobacillus* at M3-9.

When we restricted the analysis to the feces of patients who provided a sample both before and 3–9 months after the KT (paired samples from the same individual patients, Fig 4B), the relative abundance of *Lactobacilli* tended to be higher in diabetic patients than in controls at M3-9 (80-fold, p = 0.07 by Repeated Measures two-way ANOVA with Turkey’s correction). In contrast, the relative abundance of *Lactobacilli* in D0 samples did not statistically change in the three groups of patients.

*A*. *muciniphila* was detected in 50% of the controls, and 31% of all diabetic patients (non-significantly different) after transplantation. In M3-9 samples the proportion of *A*. *muciniphila* was 2,000 times lower in diabetic patients than in controls (difference of the mean Log_10_(*A*. *muciniphila*/Eubacteria) = -3.3, p = 0.002 by two-way ANOVA with Turkey’s correction, Fig 4C). The relative abundance of *A*. *muciniphila* in NODAT patients was intermediate.

In contrast, the relative abundance of *Bifidobacterium*, *Bacteroides-Prevotella*, *F*. *prausnitzii*, *E*. *coli*, *C*. *leptum* and *C*. *coccoides* and the Firmicutes/Bacteroidetes ratio were not different between NODAT patients, diabetics and controls in the M3-9 samples.

To summarize, a higher percentage of *Lactobacillus sp*. carriage, a higher relative abundance of *Lactobacillus sp*. and a lower relative abundance of *A*. *muciniphila* in the feces of the carriers characterized the gut microbiota alteration associated with diabetes and NODAT after KT.

### Changes in the composition of the gut microbiota between before and after KT

In order to evidence a correlation between a change in gut microbiota and the onset of diabetes, we compared the changes in the relative abundance of each bacterial group between D0 and M3-9 in the three metabolic groups of patients.

When analyses were restricted to paired (before and after KT) fecal samples, the relative abundance of *Lactobacilli* increased in NODAT patients and in diabetics while it remained statistically the same in controls between D0 and M3-9 (difference of the mean Log_10_(*Lactobacilli*/Eubacteria) = 1.3, *i*.*e*. a 20-fold difference in relative abundance, p = 0.06, and 1.4, *i*.*e*. a 25-fold difference, p = 0.02, and 0.2 p = 0.99 respectively by repeated measures two-way ANOVA with Turkey’s correction, Fig 4B).

Similarly, the relative abundance of *A*. *muciniphila* decreased in NODAT and in diabetic patients, while it remained statistically the same in controls between D0 and M3-9 (difference of the mean Log_10_(*A*. *muciniphila*/Eubacteria) = -3.4, *i*.*e*. a 2,500-fold decrease in relative abundance, p = 0.04, -4.7, *i*.*e*. a 50,000 decrease, p<0.0001, and -1.5, p = 0.19 respectively by two-way ANOVA with Turkey’s correction, Fig 4C).

**Fig 5:**
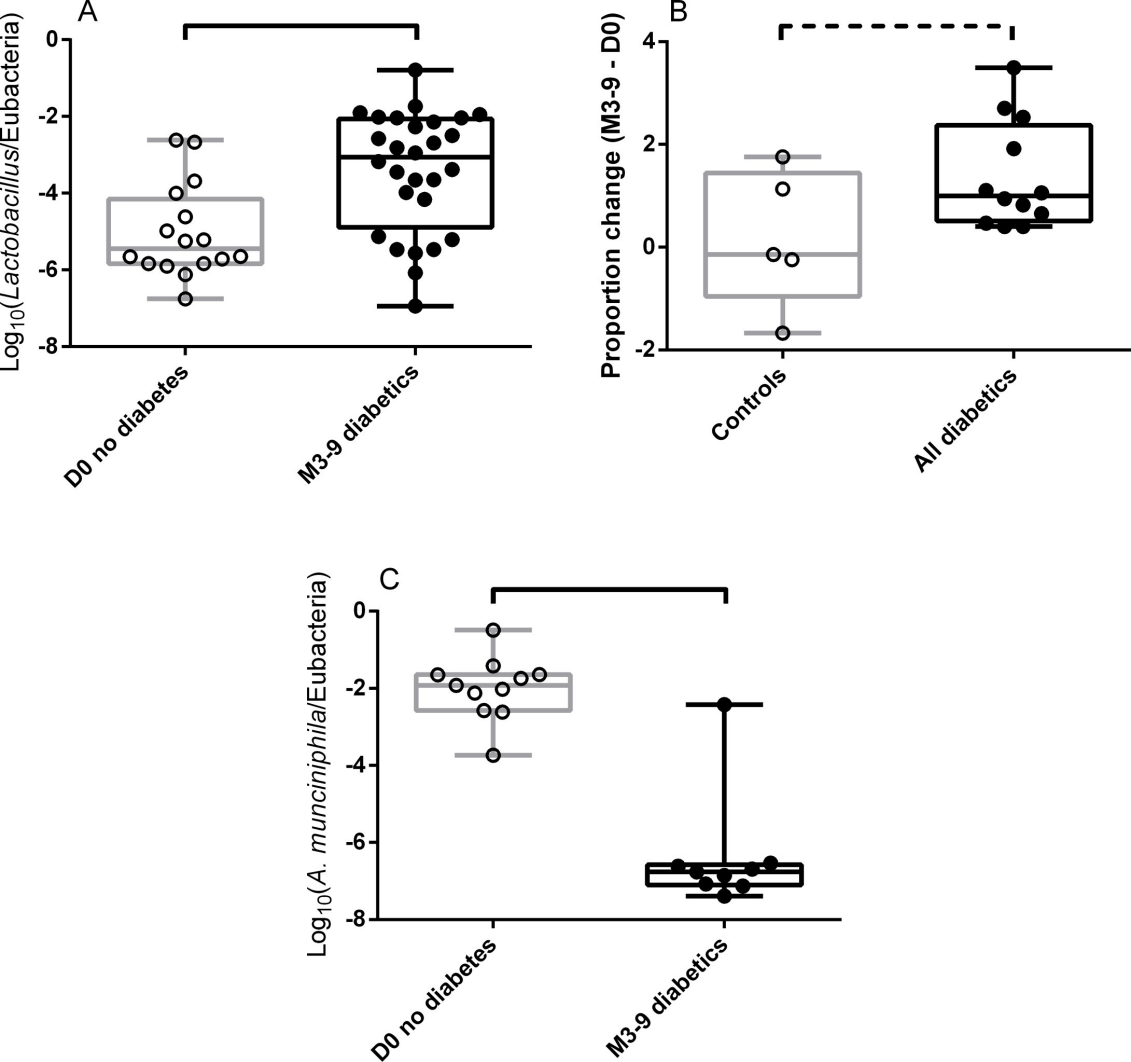
Comparisons of non-diabetic patients at D0 and diabetic patients at M3-9. A: Relative abundance of *Lactobacilli* in all non-diabetic patients at D0 (controls and NODAT patients) and all diabetic patients at M3-9 (NODAT and diabetics). B: Difference in the relative abundance of *Lactobacilli* before and after KT in the paired samples of patients without (controls) or with diabetes at M3-9 (NODAT and diabetics). While the relative abundance of *Lactobacilli* remained stable in controls, it significantly increased in all diabetics at M3-9. C: Relative abundance of *A*. *muciniphila* in all non-diabetic patients at D0 (controls and NODAT patients) and all diabetic patients at M3-9 (NODAT and diabetics). Box (25th to 75th percentiles) and whiskers (min to max) with the median (line in the middle) and all individual values (rounds) are plotted. Bars at the top of graphs indicate significant differences. Dotted bars indicate trends (p≤0.08).

The relative abundance of *Lactobacilli* (Log_10_(*Lactobacilli*/Eubacteria)) in samples from all patients without diabetes at D0 (controls and NODAT) was lower than the relative abundance of *Lactobacilli* of all patients with diabetes at M3-9 (NODAT and diabetics, -5.0±1.2 *vs*. -3.4±1.6, p = 0.0009, Fig 5A).

When we restricted the analysis to patients who provided samples at D0 and at M3-9, the difference in the relative abundance of *Lactobacilli* (Log_10_(*Lactobacilli*/Eubacteria)) between M3-9 and D0 was close to zero in controls and tended to be higher in all diabetic patients (NODAT and diabetics) at M3-9 (0.2±1.3 vs. 1.4±1.0, p = 0.06, Fig 5B).

The relative abundance of *A*. *muciniphila* (Log_10_(*A*. *muciniphila*/Eubacteria)) in samples from all patients without diabetes at D0 (controls and NODAT) was 25,000 higher than the relative abundance of *A*. *muciniphila* of all patients with diabetes at M3-9 (NODAT and diabetics, -2.0±0.8 vs. -6.4±1.5, p<0.0001, Fig 5C).

### Global effect of KT on the gut microbiota

We then evaluated whether the global process of KT influenced fecal bacterial relative abundances of included patients independently of the metabolic status at baseline.

**Fig 6:**
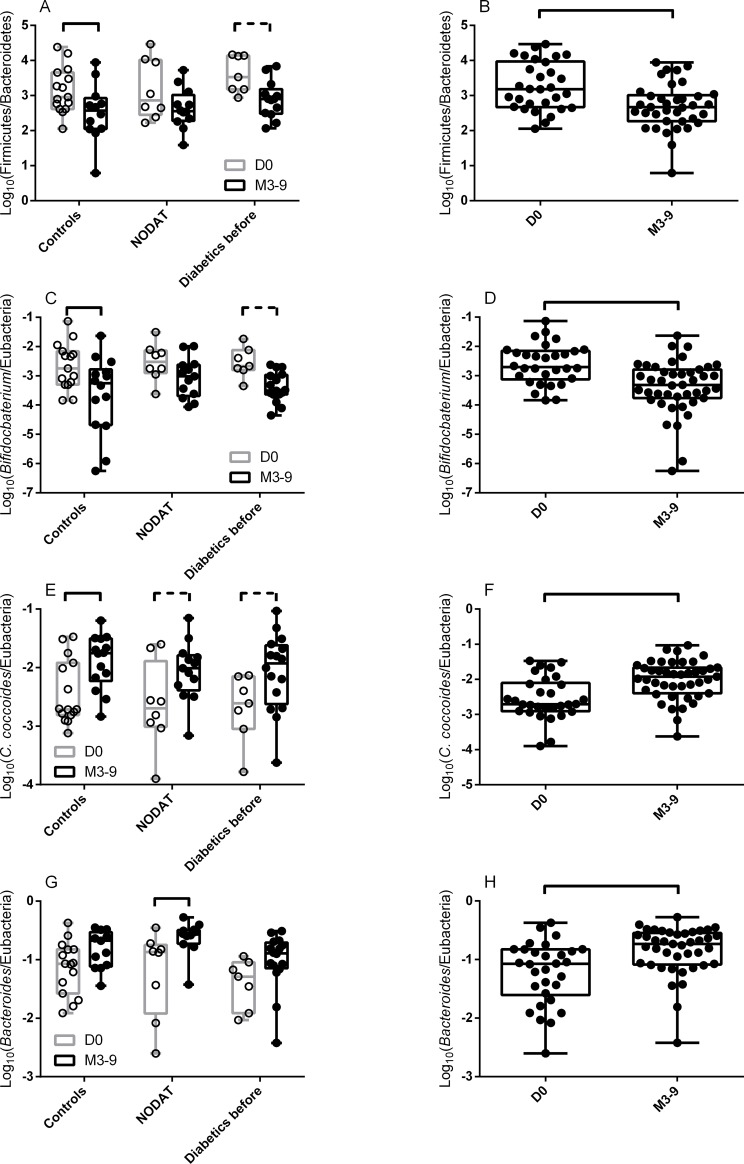
Global changes in the gut microbiota after kidney transplantation. Relative abundance of bacteria in the different metabolic groups and time points (A, C, E, G) and comparison of all D0 samples to all M3-9 samples (B, D, F, H). A, B: Firmicutes/Bacteroidetes ratio. C, D: *Bifidobacterium*. E, F: *Clostridium coccoides*. G, H: *Bacteroides*. Globally, the Firmicutes over Bacteroidetes ratio and the relative abundance of *Bifidobacterium* out of the total bacteria decreased in all metabolic groups between before and after KT. In the opposite, the relative abundance of *Clostridium coccoides* and of *Bacteroides* out of the total bacteria increased in all metabolic groups between before and after KT. Box (25th to 75th percentiles) and whiskers (min to max) with the median (line in the middle) and all individual values (rounds) are plotted. Bars at the top of graphs indicate significant differences. Dotted bars indicate trends (p≤0.08).

The Firmicutes over Bacteroides ratio decreased in all groups. This decrease was significant in controls and constituted a trend in diabetic patients (difference of the mean Log_10_(Firmicutes/Bacteroidetes): -0.6, p = 0.05 and -0.7, p = 0.07 by two-ANOVA with Turkey’s correction for multiple correction, respectively, Fig 6A). Overall, the ratio significantly decreased in all M3-9 samples compared to D0 samples (difference of the mean Log_10_(Firmicutes/Bacteroidetes): 0.6±0.2, p<0.001, Fig 6B).

Similarly, the relative abundance of *Bifidobacterium* decreased in all metabolic groups, with significance reached in controls and a trend observed in diabetic patients (difference of the mean Log_10_(*Bifidobacterium*/Eubacteria): -1.0, p<0.01 and -0.8, p = 0.08 respectively, Fig 6C). Overall, the relative abundance of *Bifidobacterium* significantly decreased in all M3-9 samples compared to D0 samples (difference of the mean Log_10_(*Bifidobacterium*/Eubacteria): -0.8±0.2, p = 0.0002, Fig 6D).

In contrast, the relative abundance of *C*. *coccoides* increased in controls and tended to increase in NODAT and diabetic patients (difference of the mean Log_10_(*C*. *coccoides*/Eubacteria): 0.5, p = 0.05, 0.6, p = 0.08, and 0.6, p = 0.06 respectively, Fig 6E). Overall, the relative abundance of *C*. *coccoides* significantly increased in all M3-9 samples compared to D0 samples (difference of the mean Log_10_(*C*. *coccoides*/Eubacteria): 0.5±0.1, p = 0.0002, Fig 6F).

Finally, the relative abundance of *Bacteroides* increased in all the metabolic groups but the increase was significant in NODAT patients only and a trend was observed in the other groups (difference of the mean Log_10_(*Bacteroides*/Eubacteria): 0.5, p = 0.05, 0.6, p = 0.08, and 0.6, p = 0.06 respectively, Fig 6G). Overall, this ratio significantly increased in all M3-9 samples compared to D0 samples (difference of the mean Log_10_(*Bacteroides*/Eubacteria): 0.5±0.1, p = 0.0002, Fig 6H).

## Discussion

We have assessed the relative abundance of specific bacteria in the feces of end-stage renal disease patients before and after KT according to their diabetic status. Despite the small number of patients, we were able to evidence bacteria whose proportions were associated with diabetes and NODAT, both before and after KT.

The gut microbiota composition alterations that we have observed are consistent with what has been described in mouse models [14–17] and in non-transplanted diabetic patients [18, 37, 38]: an increase in the relative abundance of *Lactobacillus* and a decrease in the relative abundance of *F*. *prausnitzii* and *A*. *muciniphila* in diabetics compared to controls. Furthermore, the relative abundance of *Lactobacillus sp*. increased and the relative abundance of *A*. *muciniphila* decreased between D0 and M3-9 in NODAT and in initially diabetic patients but not in controls (Fig 4). This suggests that changes in the gut microbiota composition occur with the development of diabetes, independently of whether the disease is primary or induced by medicinal drugs.

The main strength of this study is the comparison of NODAT patients to controls and initially diabetic patients as NODAT patients transition from a non-diabetic to a diabetic state throughout the study. Their diabetes (and therefore possibly their microbiota abnormalities) appear during the follow-up. Before KT, *Lactobacillus sp*. was detected at higher frequency in NODAT and diabetic patients than in controls (Fig 3A and 3B). Also, the relative abundance of *F*. *prausnitzii* was higher in controls than in diabetic patients (Fig 3C). The relative abundance of *F*. *prausnitzii* of NODAT patients was statistically indistinguishable from controls and from diabetic patients. It is therefore possible that the presence of *Lactobacillus sp*. and a lack of *F*. *prausnitzii* are components of the gut microbiota at risk for developing diabetes after KT (NODAT). We hypothesize that NODAT patients present a “predysbiosis”, *i*.*e*. an abnormal composition of their microbiota, which is associated with the development of diabetes after KT. The more precise signature (other than *Lactobacillus* carriage and *F*. *prausnitzii* relative abundance) and the predictive value of “predysbiosis” need to be studied in prospective cohorts including a larger number of patients and a complete description of the gut microbiota (using 16S rDNA sequencing or metagenomics). Indeed, we chose to quantify candidate bacteria by qPCR in this work as it was a simple and efficient way of testing the main hypothesis in a relatively small number of patients.

Overall, NODAT patients gained a significant amount of weight after KT (with BMIs of 25.9±4.6 kg/m^2^ *vs*. 27.0±4.9 kg/m^2^, p = 0.04 by repeated measures two-way ANOVA with Turkey’s correction for multiple comparisons, Fig 2), which is not surprising since obesity is a risk factor for diabetes. Interestingly, BMI also increased in initially diabetic patients after KT (26.6±3.4 kg/m^2^ *vs*. 27.9±4.3 kg/m^2^, p = 0.01, same method). Even though weight gain is a known side effect of corticosteroids and calcineurin inhibitors, not all patients undergo the same weight gain after a KT; in our study, the BMI of controls did not significantly increase. It is possible that an alteration of the gut microbiota observed after KT (Fig 5) might have participated to this weight gain and that the initial difference in gut microbiota composition could also have contributed in the difference of weight gain.

**Fig 7:**
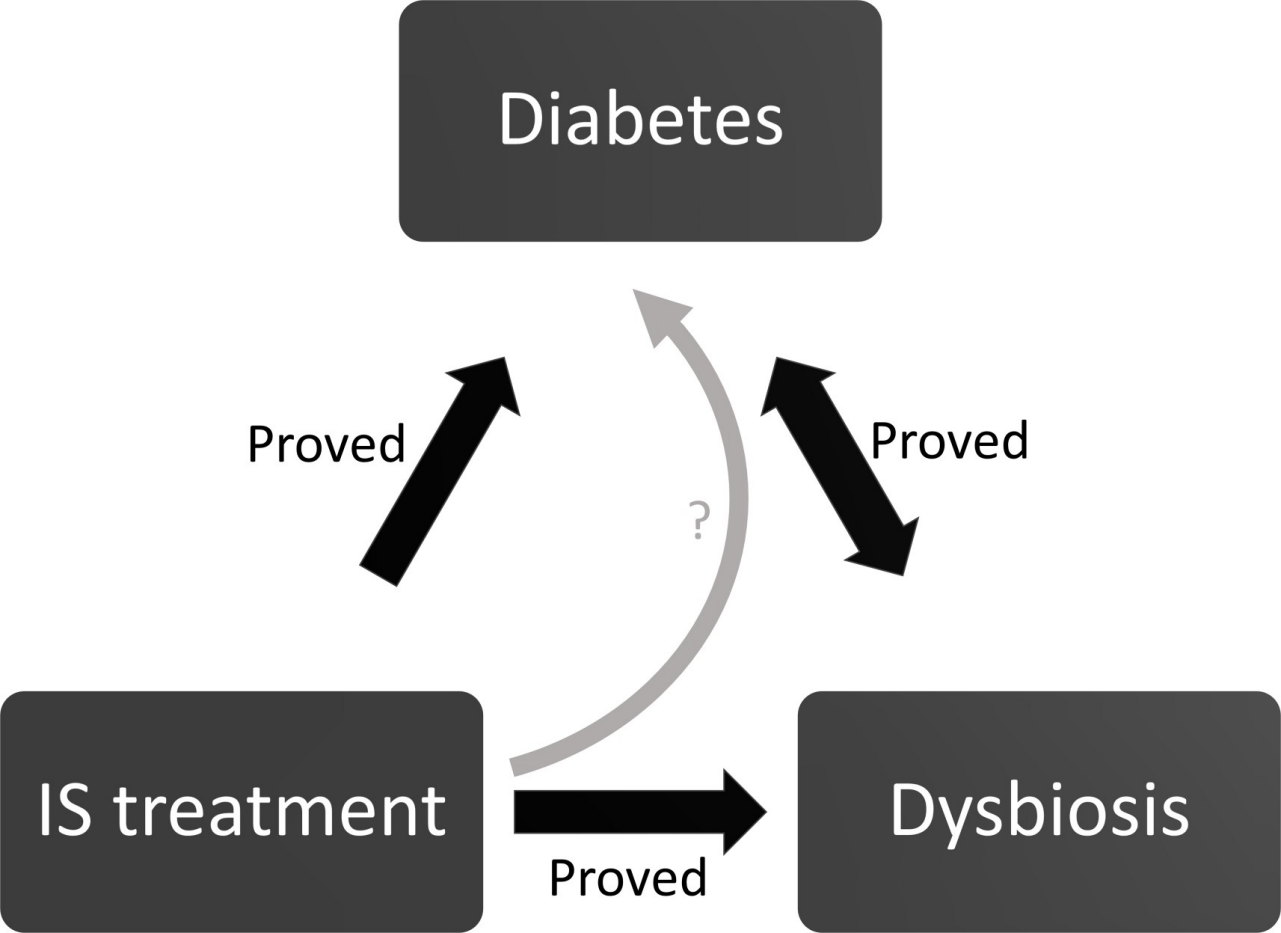
Schematic representation of the hypothetic interaction between immunosuppressive drugs, microbiota and diabetes. In this work, we hypothesize that several factors after KT, including IS treatment may induce overt dysbiosis in “predysbiotic” subjects and in turn, trigger NODAT (curved arrow with a question mark).

Altogether, we hypothesize that immunosuppressive treatment and other factors associated with KT, might aggravate a “predysbiotic microbiota”, which in turn may contribute to the development of metabolic disorders in KT patients. For example, a high relative abundance of *Lactobacillus sp*. before KT may be worsened by IS treatment and reach a degree where it contributes to the onset of NODAT (Fig 7).

If this is confirmed, it would constitute another example where the gut microbiota is involved in drug primary and/or side effects [39]. As another illustration, it is now recognized that part of the glucose-lowering effect of metformin is induced by a gut microbiota modification [40].

This study has discovered important biomarkers associated with the metabolic risks following KT. It has limits, including its retrospective design, the small number of patients included, the quantification of candidate bacteria as opposed to the full exploration of the microbiota, the absence of dietary questionnaire both before and after KT, and a wide range of delays in post-transplant fecal sample collection. Furthermore, some fecal samples had to be collected directly from anal swabbing and not by swabbing of a spontaneously emitted stool. This was due to the short delay between the patients’ arrival at the hospital and the emergency of the surgical procedure. This could possibly result in a bias in the representation of the mucosal bacteria. However, our work raises fundamental questions about the possible involvement of the gut microbiota in the development of NODAT in KTR.

## Conclusion

The role of intestinal microbiota in the onset of metabolic disorders in KT recipients needs to be further explored. The identification of a microbiota at risk for NODAT and its link with IS treatment could provide guidance towards providing the best treatment, lifestyle coaching and medical follow-up for these patients.

## Supporting information

S1 Table List of primer pairs and annealing temperatures used in this work. (DOCX)
