# Supplementary material for: Gut microbiota composition alterations are associated with the onset of diabetes in kidney transplant recipients
Source: PLoS One. 2020 Jan 7;15(1):e0227373. doi: 10.1371/journal.pone.0227373 (PMC6946168; doi:10.1371/journal.pone.0227373)
Supplement: S1 Table — (DOCX) [file pone.0227373.s001.docx]

# Supplementary table

**S1 Table: List of primer pairs and annealing temperatures used in this work.**

| **Organism/**  **Phylum** | **5'-3' Sequence** | **Annealing**  **Temperature** | **Reference** |
| --- | --- | --- | --- |
| Eubacteria | CGGTGAATACGTTCCCGG | 60 °C | [1] |
|  | TACGGCTACCTTGTTACGACTT |  |  |
| Firmicutes | CAGCAGTAGGGAATCTTC | 57 °C | [2] |
|  | ACCTACGTATTACCGCGG |  |  |
| Bacteroidetes | GCACGGGTGMGTAACRCGTACCCT | 61 °C | [2] |
|  | GTRTCTCAGTDCCARTGTGGG |  |  |
| Bacteroides | CCTWCGATGGATAGGGGTT | 60 °C | [1] |
|  | CACGCTACTTGGCTGGTTCAG |  |  |
| *F. prausnitzii* | CCATGAATTGCCTTCAAAACTGTT | 60 °C | [3] |
|  | GAGCCTCAGCGTCAGTTGGT |  |  |
| *A. muciniphila* | CAGCACGTGAAGGTGGGGAC | 63 °C | [4] |
|  | CCTTGCGGTTGGCTTCAGAT |  |  |
| *E. coli* | CATGCCGCGTGTATGAAGAA | 60 °C | [1] |
|  | CGGGTAACGTCAATGAGCAAA |  |  |
| Lactobacilli | AGCAGTAGGGAATCTTCCA | 60 °C | [1] |
|  | CGCCACTGGTGTTCYTCCATATA |  |  |
| *Bifidobacterium* | CGGGTGAGTAATGCGTGACC | 60 °C | [1] |
|  | TGATAGGACGCGACCCCA |  |  |
| *C. leptum* | CCTTCCGTGCCGSAGTTA | 60 °C | [1] |
|  | GAATTAAACCACATACTCCACTGCTT |  |  |
| *C. coccoides* | GACGCCGCGTGAAGGA | 60 °C | [1] |
|  | AGCCCCAGCCTTTCACATC |  |  |

# References

1. Furet JP, Firmesse O, Gourmelon M, Bridonneau C, Tap J, Mondot S, et al. Comparative assessment of human and farm animal faecal microbiota using real-time quantitative PCR. FEMS Microbiol Ecol. 2009;68(3):351-62. Epub 2009/03/24. doi: 10.1111/j.1574-6941.2009.00671.x. PubMed PMID: 19302550.

2. Pfeiffer S, Pastar M, Mitter B, Lippert K, Hackl E, Lojan P, et al. Improved group-specific primers based on the full SILVA 16S rRNA gene reference database. Environ Microbiol. 2014;16(8):2389-407. PubMed PMID: 25229098.

3. Sokol H, Seksik P, Furet JP, Firmesse O, Nion-Larmurier I, Beaugerie L, et al. Low counts of Faecalibacterium prausnitzii in colitis microbiota. Inflamm Bowel Dis. 2009;15(8):1183-9. Epub 2009/02/25. doi: 10.1002/ibd.20903. PubMed PMID: 19235886.

4. Everard A, Belzer C, Geurts L, Ouwerkerk JP, Druart C, Bindels LB, et al. Cross-talk between Akkermansia muciniphila and intestinal epithelium controls diet-induced obesity. Proc Natl Acad Sci U S A. 2013;110(22):9066-71. doi: 10.1073/pnas.1219451110. PubMed PMID: 23671105; PubMed Central PMCID: PMCPMC3670398.
